# Supplementary figures and images for: P2Y1 purinergic receptor identified as a diabetes target in a small-molecule screen to reverse circadian β-cell failure
Source: eLife. 2022 Feb 21;11:e75132. doi: 10.7554/eLife.75132 (PMC8860442; doi:10.7554/eLife.75132)

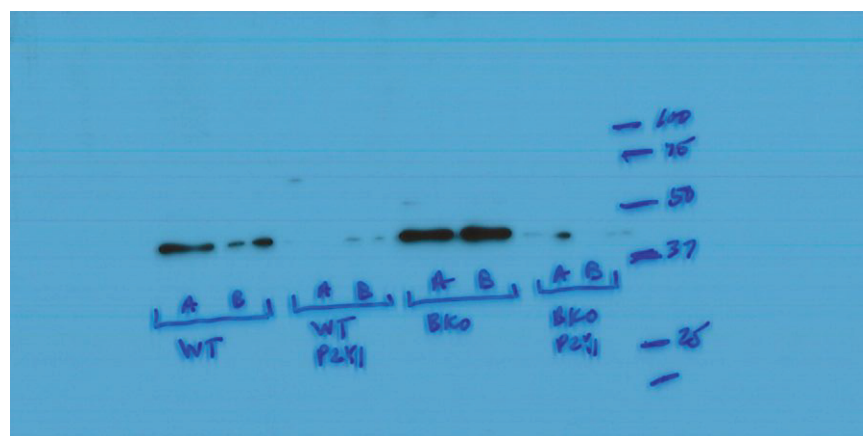

Supplement: Figure 4—figure supplement 2—source data 1. [file elife-75132-fig4-figsupp2-data1.zip › Source Data 1 Figure S4A.pdf]

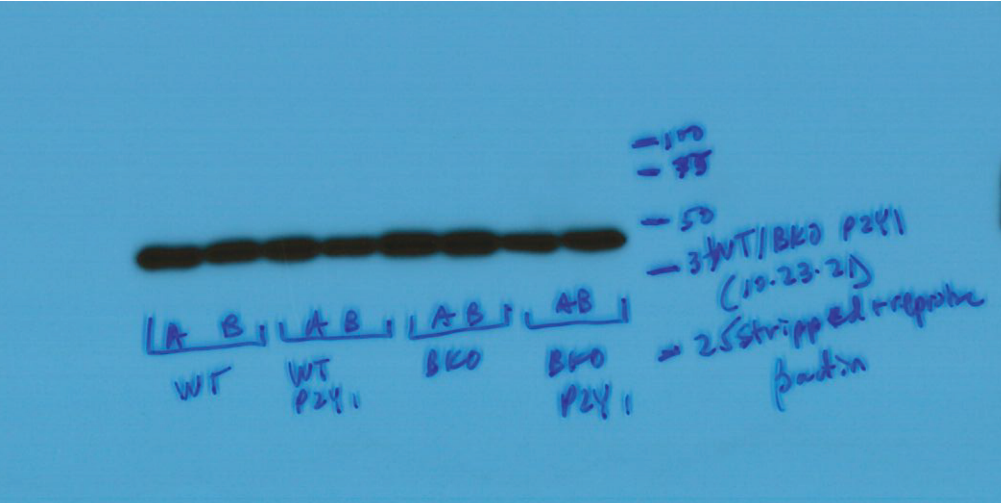

Supplement: Figure 4—figure supplement 2—source data 2. [file elife-75132-fig4-figsupp2-data2.zip › Source Data 2 Figure S4A.pdf]
